# QuickStats

**Published:** 2014-10-31

**Authors:** FROM THE NATIONAL CENTER FOR HEALTH STATISTICS

**Figure:**
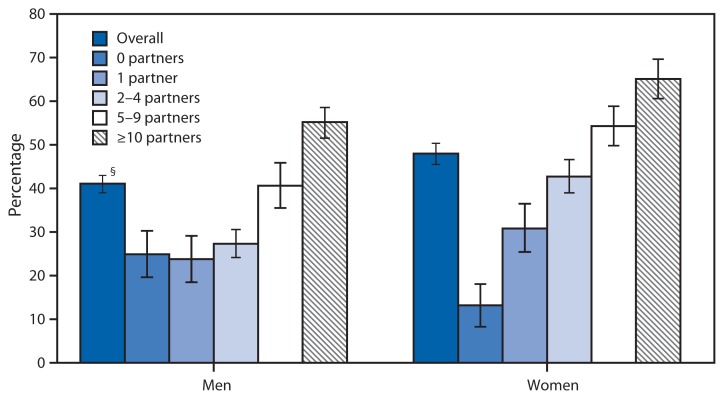
Percentage of Adults Aged 18–59 Years Who Have Ever Been Tested for HIV,*^†^ by Number of Lifetime Sex Partners and by Sex — National Health and Nutrition Examination Survey, 2007–2010 **Abbreviation:** HIV = human immunodeficiency virus. * Based on response to the question, “Except for tests you may have had as part of blood donations, have you ever had blood tested for the AIDS virus infection?” ^†^ Since 2006, CDC has recommended that all patients aged 13–64 years in any health care setting should be tested for HIV, regardless of the number of sex partners. ^§^ 95% confidence interval.

During 2007–2010, 48% of U.S. women and 41.1% of U.S. men aged 18–59 years reported having ever been tested (outside of blood donations) for HIV infection. For both men and women, an increase in the number of lifetime sexual partners increased the likelihood that they were tested for HIV. Among persons with zero lifetime sex partners, men were more likely to have had HIV testing than women (24.9% compared with 13.2%). However, among persons with 2–4, 5–9, and ≥10 lifetime sex partners, women were more likely than men to have reported any HIV testing.

**Source:** Woodring JV, Kruszon-Moran D, Oster AM, McQuillan GM. Did CDC’s 2006 revised HIV testing recommendations make a difference? Evaluation of HIV testing in the U.S. household population, 2003–2010. J Acquir Immune Defic Syndr 2014;67:331–40.

**Reported by:** Joseph V. Woodring, DO, jwoodring@cdc.gov, 301-458-4599; Deanna Kruszon-Moran, MS; Geraldine M. McQuillan, PhD; Alexandra M. Oster, MD; Steven M. Frenk, PhD.

